# Multi‐Omics Analyses Reveal Divergent Molecular Mechanisms Underlying Plant Biomass Conversion by Five Fungi

**DOI:** 10.1002/mbo3.70201

**Published:** 2025-12-18

**Authors:** Mao Peng, Jiajia Li, Li Xu, Tania Chroumpi, Sandra Garrigues, Roland S. Kun, Jiali Meng, Maria Victoria Aguilar‐Pontes, Anna Lipzen, Vivian Ng, Chaevien S. Clendinen, Nikola Tolic, Scott E. Baker, Igor V. Grigoriev, Ronald P. de Vries

**Affiliations:** ^1^ Fungal Physiology Westerdijk Fungal Biodiversity Institute Utrecht the Netherlands; ^2^ USA Department of Energy Joint Genome Institute Lawrence Berkeley National Laboratory Berkeley California USA; ^3^ Environmental Molecular Sciences Laboratory Pacific Northwest National Laboratory Richland Washington USA; ^4^ Microbial Molecular Phenotyping Group, Environmental Molecular Sciences Division, Environmental and Biological Sciences Division Pacific Northwest National Laboratory Richland Washington USA; ^5^ DOE Joint BioEnergy Institute Emeryville California USA; ^6^ Department of Plant and Microbial Biology University of California Berkeley Berkeley California USA

**Keywords:** CAZy, fungi, plant biomass conversion, sugar metabolism, sugar transporter, transcription regulation

## Abstract

Fungal plant biomass conversion (FPBC) is of great importance to the global carbon cycle and has been increasingly applied for the production of biofuel and biochemicals from lignocellulose. However, the comprehensive understanding of relevant molecular mechanisms in different fungi remains challenging. Here, we comparatively analyzed the transcriptome, proteome and metabolome profile of four ascomycetes and one basidiomycete fungi during their growth on two common agricultural feedstocks (soybean hulls and corn stover). We revealed strong time‐, substrate‐ and species‐specific responses at multi‐omics levels for the tested fungi, highlighting species‐specific carbon utilization approaches and evolutionary adaptation to environmental niches. Notably, a remarkable expressional diversity of lignocellulose degrading enzymes, sugar transporter and metabolic genes, as well as industrially relevant metabolites were identified across different fungi and cultivation conditions. The findings improves our understanding of complex molecular networks underlying FPBC and fungal ecological roles, offering novel insights that can guide future genetic engineering of fungi for valorization of agriculture waste into value‐added bioproducts.

## Graphical abstract

- Multi‐omics revealed strong time‐, substrate‐ and fungal species‐ specific responses.
- CAZymes, sugar transporter and sugar metabolic genes showed remarkable changes.
- Several valuable biocompounds were identified during fungal plant biomass conversion.
- The findings enhanced understanding of fungal lignocellulose utilization, that will facilitate biotechnology for valorization of agricultural waste.

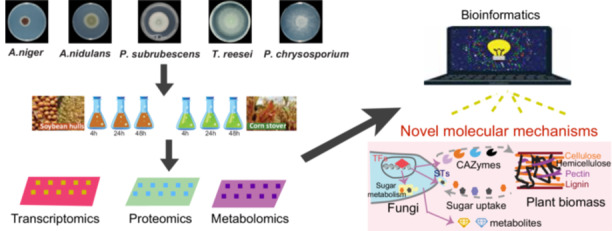

## 1 Introduction

Plant biomass is the most abundant and renewable carbon source in the world. It is mostly composed of several polymers, including cellulose, hemicellulose, pectin, and lignin, as well as protein and storage polysaccharides (e.g., starch, inulin, and gums). In nature, many fungi have developed a sophisticated system for the efficient utilization of plant materials. During the past decades, fungal plant biomass conversion (FPBC) has attracted broad research and industrial interest due to its important ecological role in the global carbon cycle, and its increasing industrial potential to produce biofuel, biochemicals and enzymes from renewable plant biomass (Meyer et al. 2020).

FPBC is a highly complex process that involves several crucial biological processes. Firstly, a broad range of extracellular enzymes are secreted to degrade the recalcitrant plant polymers. Most of these enzymes have been well cataloged in the carbohydrate‐active enzymes (CAZymes) database (www.cazy.org) (Drula et al. 2022), including glycoside hydrolases (GHs), carbohydrate esterases (CEs), polysaccharide lyases (PLs), and lytic polysaccharide monooxygenases (LPMOs). After enzymatic decomposition, plant polymers are breakdown to mono‐ and short oligosaccharides, which are transported into the cell mediated by various sugar transporters (STs) (Peng et al. 2018). Additionally, most fungi harbor a robust sugar metabolic network enabling them to convert plant‐derived sugars into other molecules to support their growth and reproduction (J. Li et al. 2022). For instance, the easily metabolizable sugars d‐glucose and d‐fructose are firstly phosphorylated before entering glycolysis. The pentoses d‐xylose and l‐arabinose are converted through the pentose catabolic pathway (PCP) to d‐xylulose‐5‐phosphate, which enters the pentose phosphate pathway (PPP) (Chroumpi, Peng, Aguilar‐Pontes, et al. 2021). Other sugars, like d‐mannose, l‐rhamnose, d‐galactose, d‐galacturonic acid and d‐gluconic acid, are converted through their individual sugar‐specific metabolic pathways (Aguilar‐Pontes et al. 2018; Claire Khosravi et al. 2015; Martens‐Uzunova and Schaap 2008) and eventually all enter glycolysis.

Previous genomics studies have revealed that the content of lignocellulolytic CAZy genes varied significantly across fungal species (de Vries et al. 2017; Riley et al. 2014; Zhao et al. 2013). Further transcriptomics and proteomics studies have demonstrated even more significant diversity regarding the production of plant biomass‐degrading enzymes in different fungi (Arntzen et al. 2020; Benoit et al. 2015; de Vries and Mäkelä 2020; Kijpornyongpan et al. 2022; Miyauchi et al. 2020; Vanden Wymelenberg et al. 2011; Wu et al. 2020). In contrast to the extensive studies of CAZymes, the systematical analysis of fungal sugar transporters and sugar metabolic networks lags behind. Only a limited set of studies have revealed the functional diversity of the sugar transporters and sugar metabolic genes during fungal growth on different monosaccharides (J. Li et al. 2022; Resl et al. 2022; L. Xu et al. 2024). Given the complex composition of plant biomass and fungal diverse approaches for carbon utilization, our current understanding of the FPBC process is still far from complete. A more comprehensive elucidation of the molecular mechanisms underlying fungal responses to different types of crude plant biomass is greatly needed for enhancing our understanding of fungal substrate preference and degradation strategies in the environment and for guiding the development of robust fungal cell factories capable of efficiently converting renewable lignocellulosic feedstocks into value‐added bioproducts.

In this study, the molecular response of five fungi to two plant residues were systematically analyzed at the transcriptome, proteome, and metabolome levels. The selected fungi include *Aspergillus niger*, *Aspergillus nidulans*, *Penicillium subrubescens*, *Trichoderma reesei*, and the white‐rot basidiomycete *Phanerochaete chrysosporium*, representing taxonomically distinct groups and are widely recognized as important model or industrial organisms. The tested substrates corn stover (CS) and soybean hulls (SBH) are common agriculture wastes, differing in sugar composition, with higher abundance of xylan and pectin in CS and SBH, respectively (C. Khosravi et al. 2019). Our study aimed to reveal complex molecular mechanisms of FPBC across diverse species, facilitating future research on exploring fungal ecological role in global carbon recycle and promoting industrial valorization of plant biomass by fungi.

## 2 Materials and Methods

### 2.1 Fungal Strains, Cultivation Media, and Growth Conditions

Five different fungi were selected to study their molecular mechanisms associated with plant biomass conversion. These include three Eurotiomycetes: *A. niger* (Aguilar‐Pontes et al. 2018), *A. nidulans* (Galagan et al. 2005), and *P. subrubescens* (Peng et al. 2017); the Sordariomycete *T. reesei* (W. C. Li et al. 2017; Martinez et al. 2004); and the Basidiomycete *P. chrysosporium* (Martinez et al. 2004), and their relevant (functional‐)genome data were analyzed.

*A. niger* N402, *A. nidulans* FGSC A4 and *P. subrubescens* CBS 132785 were pre‐cultured using complete (CM) medium (de Vries et al. 2004) with 2% d‐fructose as the sole carbon source. The same cultivation method was used for *T. reesei* QM6a and *P. chrysosporium* PR‐78, but with media optimized for these species (Eastwood et al. 2011; Klaubauf et al. 2014). Mycelia were washed with minimal medium (MM (de Vries et al. 2004)) and transferred to 250 mL Erlenmeyer flasks containing 50 mL MM supplemented with 1% corn stover (CS) or 1% soybean hulls (SBH) as carbon sources. After cultivation of fungi on these flasks for 4, 24 and 48 h, mycelia were harvested through vacuum filtration, dried between tissue paper and frozen in liquid nitrogen. Culture filtrates were also harvested and clarified for extracellular metabolomics analysis. All samples were stored at −80°C until being processed.

### 2.2 RNA Sequencing Analysis

Total RNA was extracted from ground mycelial samples using TRIzol reagent (Invitrogen, Merelbeke, Belgium) and purified with the NucleoSpin RNA Clean‐up Kit (Macherey‐Nagel, Düren, Germany), while contaminant genomic DNA was removed by rDNase treatment directly on the silica membrane. The RNA quality and quantity were analysed with a RNA6000 Nano Assay using the Agilent 2100 Bioanalyzer (Agilent Technologies, Middelburg, The Netherlands). Purification of mRNA, synthesis of cDNA library and sequencing were conducted at DOE Joint Genome Institute (JGI) as described previously (Chroumpi et al. 2020). Three biological replicates were used for each condition and the poor‐reproduced samples (Pearson's correlation coefficient of raw counts < 0.85) were discarded for further statistical analysis.

DESeq. 2 v1.20 (Love et al. 2014) was used to determine differentially expressed genes (DEGs) in comparisons of different growth conditions. Raw counts were used as DESeq.2 input. The significant DEGs (higher‐/lower‐ expressed genes) was defined based on following thresholds: fold change (FC) ≥ 4 or ≤ 0.25, adjusted *p*‐value < 0.01 (using the Benjamini–Hochberg method), and FPKM (fragments per kilobase of transcript per million fragments mapped) in at least one condition larger than 20. Besides the newly generated transcriptome data on fungi grown on crude plant biomass, transcriptome data of fungi grown on nine different monosaccharides from a previous study (J. Li et al. 2022) were also integrated and analyzed. Carbon metabolic pathways and enzymes were extracted from our previous study (J. Li et al. 2022). The CAZy family information was obtained from JGI MycoCosm database (Grigoriev et al. 2014), and annotation of plant polysaccharide specificity of CAZymes and STs was based on previous publications (Benoit et al. 2015; Daly et al. 2018; L. Xu et al. 2025). Heatmap and clustering were generated using R package “ComplexHeatmap” (Gu et al. 2016). The principal component analysis (PCA) was performed with the DEseq.2 (Love et al. 2014), using the normalized counts by applying the *regularized log (rlog)* method. Gene set variation analysis (GSVA) (Hänzelmann et al. 2013) was performed to estimate variation of gene set activity over the tested conditions, focusing on the genes encoding preselected CAZy, sugar transporters, and sugar metabolic enzymes. The average values of gene expression (FPKMs) in each studied condition were used as input for GSVA analysis. The RNA‐seq data generated in this study are available at the NCBI SRA database, with accession IDs: SRP369472–SRP369500 for *P. subrubescens*; SRP448957–SRP448974 for *A. niger*; SRP448919–SRP448936 for *A. nidulans*; SRP448939–SRP448956 for *T. reesei*; and SRP369438–SRP369455 for *P. chrysosporium*.

### 2.3 Bioinforimatics Identification and Phylogenetic Analysis of Sugar Transporters

STs were extracted from our previous study (L. Xu et al. 2025). Protein sequences of STs were aligned using Muscle v5.3 (Edgar 2022). The positions that contained > 20% gaps were removed from alignment using trimAl (Capella‐Gutiérrez et al. 2009). The phylogenetic analysis was performed with maximum likelihood method using IQ‐TREE v2.4.0 (Minh et al. 2020) with 1000 UFBoot2 bootstrapping and the best‐fit model determined by ModelFinder. The other parameters of IQ‐TREE were set as default. One characterized rhamnose ST from *Aspergillus niger* (Sloothaak et al. 2016), which belongs to the major facilitator superfamily (PF07690) but not the sugar transporter family (PF00083), was used as out‐group in the phylogenetic analysis for rooting the tree. Sugar specificity of predicted STs was assigned through incorporating the characterized STs in the phylogenetic tree based on the assumption that the genes of the same clade tend to share similar function (Eisen and Wu 2002). The the phylogenetic tree was visualize using online tool iTOL (Letunic and Bork 2016).

### 2.4 Proteomic Analysis

The sample preparation and proteomic analysis of intracellular proteins from mycelia were performed using similar approach as previously described (Daly et al. 2020). Briefly, the intracellular proteome was analyzed using equal amounts of proteins from each sample. Mass spectrometry (MS) analysis was performed using a Q‐Exactive Plus mass spectrometer (Thermo Scientific, USA) outfitted with a homemade nano‐electrospray ionization interface. The ion transfer tube temperature and spray voltage were 300°C and 2.2 kV, respectively. Data were collected for 120 min following a 10 min delay after completion of sample trapping and start of gradient. Fourier‐transform (FT)‐MS spectra were acquired from 300 to 1800 m/z at a resolution of 70 k (AGC target 3e6), and the top 12 FT‐HCD‐MS/MS spectra were acquired in data‐dependent mode with an isolation window of 1.5 m/z at a resolution of 17.5 k (AGC target 1e5) using a normalized collision energy of 30, dynamic exclusion time of 30 s, and detected charge state of an ion 2 to 8. Generated MS/MS spectra were searched against protein sequences of each fungus obtained from MycoCosm database using (MSGF+ ) (Kim and Pevzner 2014). Best matches from the MSGF+ searches were filtered at 1% FDR, and MASIC software was used to pull abundances for identified peptides (Monroe et al. 2008). Only protein‐specific peptides (peptides unique to protein in the whole protein collection) were used in consequent analysis and aggregation. InfernoR software (Polpitiya et al. 2008) was used to transform peptide abundances (log2) and perform mean central tendency normalization. Protein‐grouped normalized peptide abundances were de‐logged, summed, transformed (log2) and normalized again in InfernoR to produce normalized abundances for the protein‐level roll‐up. Protein abundances were then filled with zeros for missing values. The proteins that have been identified in at least three samples were further analyzed. The nonzero abundances from the three replicates of different growth conditions were compared with *T*‐test. Significant differentially expressed proteins were selected based on threshold of fold change (FC) ≥ 2 or ≤ 0.5 and *p*‐value < 0.01. The mass spectrometry proteomics data have been deposited to the ProteomeXchange Consortium via the MassIVE partner repository with the data set identifier MSV000098008.

### 2.5 Metabolomic Analysis

Metabolomic analysis of intracellular and extracellular metabolites was performed as previously described (Chroumpi, Peng, Markillie, et al. 2021). Briefly, metabolites were extracted from the ground mycelia or culture filtrates, which underwent methoximation and silylation with N‐methyl‐N‐trimethylsilyltrifluoroacetamide and 1% trimethylchlorosilane (MSTFA). Derivatized samples were analyzed using an Agilent GC 7890 A, coupled with a single quadrupole MSD 5975 C (Agilent Technologies, USA) with a standard mixture of fatty acid methyl esters (FAMEs) for RI alignment. GC‐MS raw files were processed and analyzed using Metabolite Detector software, version 2.5 beta (Hiller et al. 2009). The metabolites that have been identified in at least three samples were further analyzed. The significant differentially expressed metabolites were selected based on threshold of fold change (FC) ≥ 2 or ≤ 0.5 and *p*‐value < 0.01 based on T‐test. The GC/MS data have been deposited to the MassIVE database with accession number MSV000098008.

## 3 Results and Discussion

### 3.1 Transcriptome and Proteome Analysis Revealed Strong Condition‐ and Species‐Specific Responses

**Figure 1:**
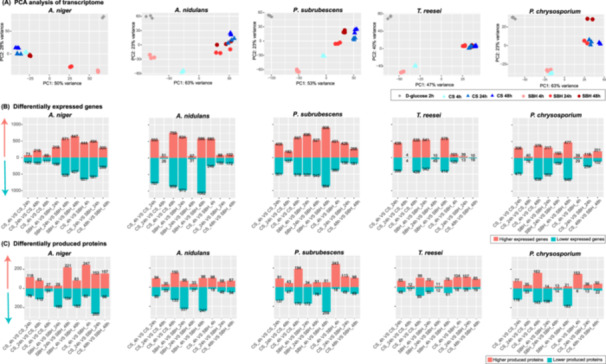
Comparisons of transcriptome and proteome profiles of five fungi. (A) Principal component analysis (PCA) of transcriptome data. PCA was performed on normalized counts of the full transcriptome of each species. Samples grown on different carbon sources (glucose, corn stover (CS) and soybean hulls (SBH)) at different time points are indicated with different shapes and colors. (B) Total number of significantly higher (in red) and lower (in cyan) expressed genes identified in each comparison. (C) Total number of significantly higher (in red) and lower (in cyan) produced proteins identified in each comparison.

Transcriptomes of five fungi (*A. niger, A. nidulans, P. subrubescens, T. reesei*, *P. chrysosporium*) grown on two plant substrates (CS, SBH) at three time points (4, 24, 48 h) were comparatively analyzed. The PCA (Figure 1A), and differentially expressed genes (DEGs) and differentially produced proteins (DPPs) (Figures 1B,C, respectively) revealed that fungal response to plant biomass showed a strong substrate‐, time‐ and species‐specific patterns. Compared to gene expression profiles on the easily metabolizable d‐glucose, all studied fungi showed significantly different transcriptome response to crude plant biomass (CS and SBH) (Figure 1A). Moreover, a considerable diversity of their response to CS and SBH was observed at both transcriptome (Figure 1B and Supporting Information S2: Table S1) and proteome levels (Figure 1C and Supporting Information S2: Table S2). The DEGs identified from *A. niger* grown on CS at different time points showed only a minimal difference, while their expression on SBH showed a clear time‐specific response. For *A. nidulans*, the major difference in transcriptome and proteome response to the two tested plant biomass substrates was identified at 4 h, while similar profiles were observed at the later time points (24 and 48 h). *P. subrubescens* displayed a strong substrate‐ and time‐ specific response, with a considerable set of DEGs and DPPs identified in each comparison. *T. reesei* showed a profile similar to that of *A. nidulans*, with significantly different profiles at 4 h and only small sets of DEGs and DPPs detected at two later time points when comparing the two plant substrates. *P. chrysosporium* presented a minimal substrate‐specific transcriptome response at 4 h compared to the other four species, in which only 68 DEGs were detected, while 159 DPPs were identified in the corresponding comparison. In contrast, a clear differential transcriptome and proteome response of *P. chrysosporium* was identified between 4 h and the later time points (24 or 48 h) for both plant biomass substrates.

### 3.2 Expression Changes of Key Genes Associated With Plant Biomass Conversion in Five Fungi

Four important biological processes and relevant genes have been well‐documented to play crucial roles during FPBC. These include the CAZymes involved in lignocellulose degradation, sugar transporters (STs) mediating transmembrane uptake of small sugars, sugar metabolism (SM) enzymes for intracellular metabolism of sugars, and transcription factors (TFs) that regulate the expression of key genes (Peng et al. 2021). The detailed annotation of CAZymes, STs, SM and TFs genes of the tested fungi were listed in Supporting Information S2: Table S1, and comparisons of their transcriptome and proteome profiles were discussed below.

#### 3.2.1 Transcriptome Profile of Plant Biomass Degrading CAZy Genes in Five Fungi

**Figure 2:**
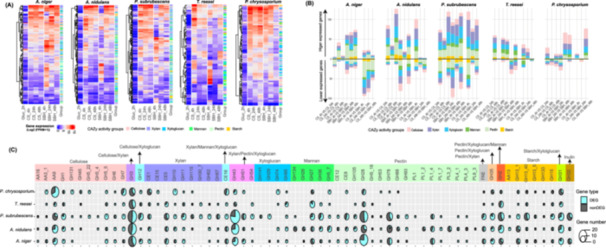
Detailed transcriptome profile of plant biomass degrading (PBD) CAZy genes in five fungi. (A) Heatmap showing the expression profile of PBD CAZy genes in each species. (B) Total number of differentially expressed genes (DEGs) identified in comparison of fungi grown on different substrates at different time points. The number of genes involved in the decomposition of different plant polymers are indicated in different colors. (C) Distribution of total and substrate‐specific expressed genes from each CAZy family across five fungi. The total number of genes from each CAZy family in each species is indicated with individual circles in different size, while the cyan part of each circle indicates the fraction of differentially expressed CAZy genes between two substrates at ≥ 1 time points.

The expression of plant biomass degradation (PBD)‐related CAZy genes were significantly induced in all five fungi during their growth on the crude plant substrates compared to glucose (Figure 2A), while their detailed changes across different time points and substrates showed strong species‐specificity (Figures 2B,C). Even though the genomes of two taxonomically close fungi, *A. niger* and *A. nidulans*, encode a comparable set of PBD‐related CAZy genes, these species showed distinct transcriptome profiles (Figure 2C). For instance, a much higher number of pectinolytic genes (e.g., from GH28, GH78 and CE12) of *A. niger* were higher expressed on SBH than CS, while only a small part of these genes showed a similar expression pattern in *A. nidulans* (Figure 2C).

For *A. niger*, the expression profile of the PBD‐related CAZy genes showed a much less time‐dependent profile on CS compared to SBH (Figure 2B). A considerable number of CAZy genes involved in xylan, xyloglucan, pectin, and starch degradation were more highly expressed on SBH than on CS, while specific temporal changes were also observed during its growth on SBH. Notably, most CAZy genes of *A. niger* were higher expressed at 4 or 24 h than at 48 h during growth on both substrates. Although cellulose is abundantly present in both CS and SBH, a relatively small number of cellulolytic genes were differentially expressed during growth of *A. niger* on these substrates compared to other studied fungi (except *T. reesei*) (Figure 2B,C).

For *A. nidulans*, a much larger number of PBD‐related CAZy genes showed higher expression at the early time point (4 h) than 24 or 48 h on CS, while comparable higher and lower expressed genes were identified in comparison of 4 h and the two later time points on SBH (Figure 2B). Much less CAZy genes were identified as DEGs in the comparison between 24 and 48 h on both plant substrates, which is in line with similar global expression profiles observed between these two time points (Figure 1A,B). Unlike *A. niger* in which most PBD‐related CAZy genes showed higher expression on SBH than CS, a larger number of *A. nidulans* genes related to degradation of cellulose, xylan, xyloglucan and mannan were higher expressed in CS than SBH at 4 h (e.g., AA9, GH3, GH43, GH95 and GH5_7 families), while much less DEGs were identified in comparison of later time points (Figure 2B,C).

*P. subrubescens* tailored its expression of PBD‐related CAZy genes continuously for both plant substrates. The genes involved in degradation of cellulose, xyloglucan, xylan, starch and pectin showed strong temporal expression profiles on both substrates (Figure 2B). The comparison between CS and SBH showed that CS induced more genes related to degradation of xylan, cellulose, and xyloglucan at all time points, while SBH induced more genes involved in starch and mannan degradation at 4 h.

*T. reesei* possesses a smaller number of genes involved in PBD (J. Li et al. 2023; Martinez et al. 2008). Growth of *T. reesei* on different conditions identified less differentially expressed PBD genes compared to the three Eurotiomycetes (Figure 2B). The PBD‐related CAZy genes of *T. reesei* were mainly induced at 4 h during its growth on CS, while small number of genes involved in degrading xylan, cellulose and mannan were induced at 48 h compared to 4 or 24 h on SBH. Compared to growth on SBH, growth on CS induced more cellulolytic and xylanolytic genes at 4 and 24 h, while some mannanolytic genes (e.g., GH27 and GH36) were specifically induced on SBH at 4 h (Figure 2B,C).

For *P. chrysosporium*, a relatively small set of PBD‐related CAZy genes were differentially expressed compared to other species (Figure 2B), except for a higher number of genes from AA9, GH10 and GH74 (Figure 2C). During its growth on CS, higher expression of cellulolytic genes was observed at 4 and 48 h compared to 24 h. In contrast, during its growth on SBH, most PBD genes showed low expression at 48 h (Figure 2A). The comparison between CS and SBH identified small sets of PBD genes differentially expressed at 4 and 24 h, but a considerable number of genes related to xylan, cellulose and xyloglucan genes showed significantly higher expression on CS than SBH at 48 h.

Overall, the diverse expression profile of PBD‐related CAZy genes across studied species reflected the polysaccharide composition of plant substrates, as well as species‐specific carbon preference. For instance, the higher abundance of xylan in CS induced a larger number of xylanolytic genes during growth on CS than on SBH for most of the studied fungi (except *A. niger*). Although SBH contains more pectin than CS, only pectinolytic genes of *A. niger* showed clear induction by this polysaccharide (Figure 2B). In line with the relatively poor growth of *A. niger* on cellulose and less efficient utilization of pectin for *P. chrysosporium* (J. Li et al. 2023), a small set of actively expressed cellulolytic and pectinolytic genes was identified during growth of *A. niger* and *P. chrysosporium* on CS (cellulose enriched) and SBH (pectin‐enriched), respectively.

#### 3.2.2 Gene Expression Profile of Sugar Transporters

**Figure 3:**
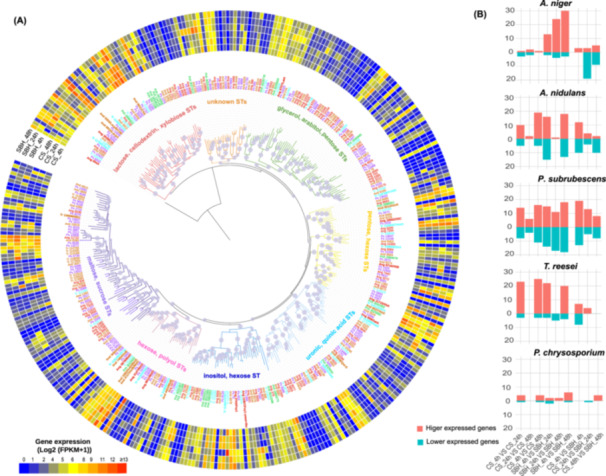
Detailed transcriptome profile of sugar transport (ST) genes in five fungi. (A) Expression and phylogenetic profiles of ST genes from five species. Expression levels from low to high are indicated by blue to red. Sugar specificity of each STs clade is labeled next to gene IDs and corresponding branches are shown in specific colors. The gene IDs of STs from different species are shown in different colors (red for *Aspergillus niger*, orange for *A*. *nidulans*, purple for *Penicillium subrubescens*, cyan for *Trichoderma reesei*, and green for *Phanerochaete chrysosporium*)and the experimentally characterized genes are shown in bold fonts. (B) Total number of significantly higher and lower expressed ST genes in each comparison, shown in red and cyan, respectively.

Using a computational analysis of the conserved ST domain (L. Xu et al. 2024), 90, 83, 117, 52, and 23 STs for *A. niger*, *A. nidulans*, *P. subrubescens*, *T. reesei* and *P. chrysosporium* were identified, respectively. The diversity of STs was not only reflected in the genomes of the selected fungi, but could also be seen in the transcriptome. Although the genomes of the three Eurotiomycetes encode much larger sets of STs than two other selected species, around half of these STs showed very low expression (FPKM < 10) in all tested conditions, while most STs from *T. reesei* and *P. chrysosporium* showed moderate to high expression (Figure 3A). In general, the STs showed similar expression trends as the CAZy genes for the studied fungi (Figures 3B and 2B, Supporting Informatiopn S1: Figure S2C) that support coordination between release of small sugars from enzymatic saccharification of lignocellulose and their transmembrane uptake. For instance, much more STs of *A. niger* showed higher expression on SBH than on CS, such as the well‐characterized *gatA* (Sloothaak et al. 2014) and *mstA* (Vankuyk et al. 2004) involved in transport of d‐galacturonic acid and hexose, respectively. A small set of STs showed expression changes during growth of *A. nidulans* and *T. reesei* on plant substrates at 24 and 48 h. Notably, *P. subrubescens* tailored the expression of specific sets of STs at each tested condition, while only few STs of *P. chrysosporium* showed significant expressional change across all conditions.

#### 3.2.3 Transcriptome Profile of Genes Involved in Sugar Metabolism

**Figure 4:**
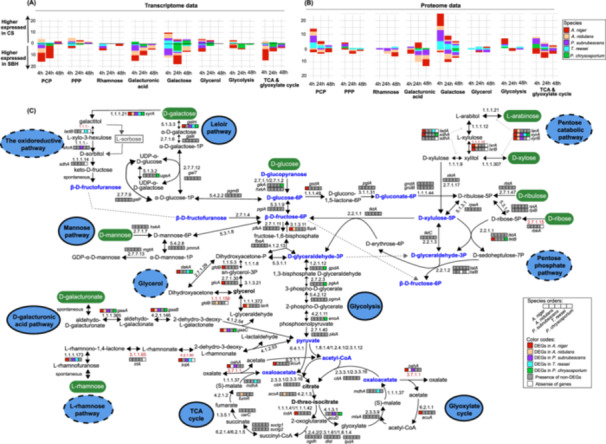
Transcriptome and proteome profiles of sugar metabolic (SM) genes/proteins in five fungi. (A) Total number of significantly higher and lower expressed SM genes for each specific SM pathway that have been identified from comparative transcriptome analysis of each species. (B) Total number of significantly higher and lower produced SM enzymes for each specific SM pathway that have been identified from comparative proteome analysis of each species (C) The expressional difference of predicted SM genes between fungi grown on two substrates. Each gene name and related expression pattern are shown next to corresponding enzymatic reactions in the sugar metabolic network. The DEGs from different species are shown in specific colors, while the genes with no significant change between two substrates are shown in grey and the ortholog gene missing in specific species are shown in white.

The comparison of gene and protein profiles related to sugar metabolism (SM) also revealed clear diversity across the studied fungi (Supporting Information S1: Figure S1, Figure 4A,B). Overall, the transcriptome profile of SM genes showed a similar pattern as the PBD‐related CAZy genes, with some exceptions observed in *P. subrubescens* and *P. chrysosporium* (Supporting Information S1: Figure S2A,B). Consistent with the enrichment of d‐galacturonic acid in SBH (C. Khosravi et al. 2019), the d‐galacturonic acid metabolic genes showed significantly higher expression on SBH than CS for most of the studied fungi (except *T. reesei*) (Figure 4A,C).

More specifically, a higher number of *A. niger* SM genes showed a more dynamic changing expression profile on SBH compared to CS (Supporting Information S1: Figure S2B). The genes involved in tricarboxylic acid (TCA) cycle, PCP, d‐galacturonic acid pathway and d‐galactose metabolism showed striking higher gene expression on SBH than CS (Figures 4A,C), while few SM enzymes related to glycolysis, d‐galacturonic acid and d‐galactose metabolism showed higher expression on proteomics data of CS at 4 or 24 h (Figure 4B).

For *A. nidulans*, similar as for the PBD‐related CAZy genes, more differentially expressed SM genes were identified in comparison of early (4 h) and later time points (24 or 48 h) than comparing two later time points (Supporting Information S1: Figure S2B). The comparison of time points on both substrates showed that SM genes related to the PCP and d‐galacturonic acid pathway were predominantly induced at 4 h, while genes related to glycolysis and glycerol metabolism were mainly induced at 24 and 48 h. Both comparisons of transcriptome and proteome between the two substrates showed that the d‐galacturonic acid pathway is more active on SBH than CS, while the d‐galactose pathway showed earlier induction for CS (at 4 h) than for SBH (at 24 or 48 h) (Figure 4A,B).

The comparison of *P. subrubescens* showed slightly different expression patterns compared to the trend of the CAZy profile discussed above. For instance, SM genes were mainly induced at 4 h (Supporting Information S1: Figure S2B), while a comparable number of CAZy genes were specifically induced at both early and later time points (Figure 2B). The most obvious transcriptome changes between *P. subrubescens* grown on CS and SBH occurred at 4 h, with a significant higher number of SM genes involved in the metabolism of PCP, d‐galacturonic acid and d‐galactose showed higher expression on SBH than on CS (Figure 4A and Supporting Information S1: Figure S2B). In contrast, some PCP and d‐galactose metabolic enzymes showed higher protein expression on CS at 4 h, indicating complex regulation at levels of transcripts and proteins for these SM genes (Figure 4B).

The SM genes of *T. reesei* during growth on both plant substrates showed a similar global expression pattern as observed for *P. subrubescens*, in which most genes were predominantly induced at 4 h and only few genes were differentially expressed between 24 h and 48 h (Supporting Information S1: Figure S2B). The DEGs and DPPs identified in comparison between CS and SBH are mainly involved in PCP, d‐galactose and l‐rhamnose pathways (Figure 4A,B).

*P. chrysosporium* has a smaller number of SM genes/proteins differentially expressed compared to other tested species, except that during growth on SBH at 24 h a significant larger number of SM genes were induced than in the other conditions (Figure 4A,B Supporting Information S1: Figure S2B). In contrast, the PBD‐related CAZy genes were mainly differentially expressed at 48 h in comparison fungal growth on CS and SBH (Figure 2B).

Overall, the DEGs and DPPs of SM genes identified in transcriptome and proteome comparisons, respectively, revealed limited consistence and large variation (Figures 4A,B, Supporting Information S1: Figure S2B,D). For instance, similar small numbers of DEGs and DPPs between comparison of two later time points in *A. nidulans* and *T. reesei* were identified. d‐galacturonic acid metabolic enzymes in most tested fungi showed higher expression at both transcript and protein levels in SBH than CS, matching the composition of this sugar in two tested plant substrates. However, the detailed response to plant substrate of the five species showed remarkable variation between DEGs and DPPs. The limited correlation between transcriptome and proteome data indicates additional regulation mechanisms after gene transcription (e.g., translation, protein stability, and post‐translational modifications (PTMs)) could affect the intracellular abundance and activity of the SM enzymes (Vogel and Marcotte 2012). In yeast, various PTMs, such as phosphorylation and acetylation have been identified in many metabolic enzymes and were suggested to modulate their activity and stability (Henriksen et al. 2012; Oliveira et al. 2012; Tripodi et al. 2015).

#### 3.2.4 Transcriptional Coordination of FPBC Gene Sets and Carbon Preference in the Five Fungi

**Figure 5:**
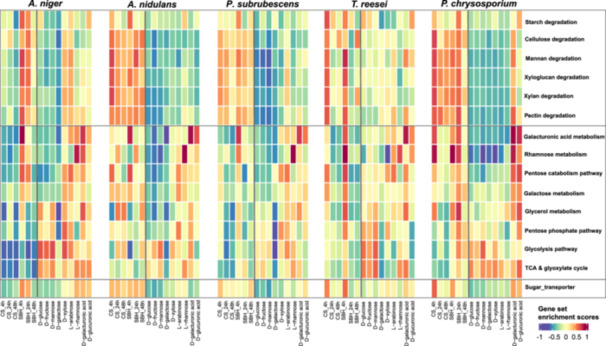
Gene set variation analysis of FPBC‐related gene sets in transcriptome data of five fungi during their growth on two crude plant biomass and nine different monosaccharides. The gene sets of CAZy, sugar metabolism, and sugar transport are shown in different boxes. Expression activity of each gene set from low to high is indicated with colors from dark purple to red.

To further reveal how the PBC‐related CAZy, sugar transporters and SM genes are transcriptionally coordinated in different carbon sources, their overall expression activities across a large transcriptome dataset were compared. RNA‐seq data of fungi grown on two crude plant biomass from this study and nine monosaccharides from a previous study (J. Li et al. 2023) were combined for analysis. The variation of expression activity of specific gene sets over the tested conditions was estimated by Gene Set Variation Analysis (GSVA) in an unsupervised manner (Hänzelmann et al. 2013) (Figure 5). In general, the GSVA results not only revealed similar strong substrate‐ and time‐ specific transcriptome changes for most of the FPBC gene sets as reported above, but also uncovered diverse response to different monosaccharides and possible association with carbon preferences across species. For instance, overall higher expression levels of genes encoding CAZymes, STs, and SM enzymes were identified on SBH at 4 h for *A. niger*; on both CS and SBH at 4 h for *T. reesei*; and on CS at 4 h and SBH at 4 and 24 h for *P. chrysosporium*. The polysaccharide degrading CAZy genes showed higher expression on crude plant biomass than on monosaccharides for most of the tested fungi, especially *A. nidulans*, *P. subrubescens* and *P. chrysosporium*. In contrast, many SM genes could be significantly induced by their corresponding monosaccharides. For instance, the TCA and glycolysis of all five species showed higher expression on easily metabolizable sugars, that is, d‐glucose, d‐mannose and d‐fructose. The genes related to metabolism of pentose, d‐galacturonic acid and l‐rhamnose showed higher expression levels on corresponding sugars, respectively. One exception is that many SM, CAZy and ST genes of *P. chrysosporium* were nonspecifically induced by d‐galacturonic acid and d‐glucuronic acid (Figure 5).

The transcriptional diversity of FPBC genes across tested fungi could be linked to their environmental adaptation. Many cellulolytic and hemicellulolytic CAZy genes of *A. niger* showed higher expression during growth on two pentoses (d‐xylose and l‐arabinose). The pectinolytic CAZy genes of *A. niger* were mainly induced on d‐galacturonic acid, l‐rhamnose and l‐arabinose that are three main sugar components of pectin and inducers of the pectinolytic transcriptional activators GaaR (Alazi et al. 2016), RhaR (Gruben et al. 2014) and AraR (Battaglia et al. 2011), respectively. In contrast, the CAZy genes of other species were less significantly induced by d‐xylose and l‐rhamnose. Compared to other species, FPBC genes of *A. niger* showed poor expression on CS, which matches its slow growth on this substrate (C. Khosravi et al. 2019). The strong induction of pectin‐ related degrading genes and metabolic genes at 4 h of SBH suggests a clear pectin‐favored approach for *A. niger*. The differences between *A. niger* and *A. nidulans* on SBH mirror those observed for another dicot feedstock (sugar beet pulp), in which a proteomic analysis revealed a higher production of cellulases for *A. nidulans*, while *A. niger* produced mainly pectinases (Mäkelä et al. 2018). Interestingly, *P. subrubescens* produced highly similar enzyme activities as *A. niger* on SBH (Mäkelä et al. 2016), while in our study, it was more similar to *A. nidulans*. This suggests that culture conditions and sample times strongly affect fungal gene expression. The lower induction on d‐xylose than on l‐arabinose for CAZy genes in *P. subrubescens* supports the dominant role of the l‐arabinose responsive TF AraR in controlling pentose utilization in this fungus (Liu et al. 2024). The basidiomycete *P. chrysosporium* showed less specific transcriptome response to SBH and CS, which could be explained by CS and SBH being non‐natural substrates for this fungus. *P. chrysosporium* naturally grows on woody substrates that have a different composition (Martinez et al. 2004). Additionally, *P. chrysosporium* showed a similar response to d‐galacturonic acid and d‐glucuronic acid. While the first compound is a main component of pectin that is only marginally present in wood, the second is a common composition in side chain of wood xylans (Curry et al. 2023). This suggests that the enzymatic induction system in this species does not distinguish between these two uronic acids.

#### 3.2.5 Diverse Transcriptome Profile of Transcription Factors Across Five Fungi

The dynamic change of gene expression is driven by TFs. Previous studies have reported a diverse set of key TFs related to FPBC across different species (Benocci et al. 2017), and the expression profiles of these TFs (including experimentally characterized and ortholog‐based predicted TFs) have been comparatively analyzed here. In contrast to the larger set of TFs of Ascomycetes, only three FPBC‐associated TFs have so far been reported in Basidiomycetes (i.e., *cre1* (Pareek et al. 2022), *pacC* (Zhu et al. 2022), and *ace3*/*roc1* (Benocci et al. 2017; Marian et al. 2022)). As expected, the expression profile of these TFs showed remarkable diversity (Supporting Information S1: Figure S3). For instance, the expression of highly conserved carbon catabolic repressor *creA/cre1* showed high expression in all the tested conditions for the three Eurotiomycetes, while its expression was only induced by few tested conditions for *T. reesei* and *P. chrysosporium*. Another conserved regulator, the pH‐responsive TF, *pacC*, showed a similar expression pattern, which was slightly induced by uronic acid and SBH conditions for all four Ascomycetes, while its expression is constantly low for *P. chrysosporium*. Additionally, the expression profile of a crucial TF controlling pectin utilization, *gaaR* (Alazi et al. 2016), showed higher expression on d‐galacturonic acid, and on SBH at 4 and 24 h for *A. niger*, and higher expression on CS at 4 h for *A. nidulans*, while transcriptional variation was much lower for the other Ascomycetes. The AmyR gene regulating starch utilization showed higher expression on SBH at 4 h for *A. niger*, *A. nidulans* and *T. reesei*, while its expression was only slightly induced on SBH at 48 h for *P. subrubescens*, indicating different carbon preference among these fungi. The ace3 gene has been characterized for regulating cellulose degradation in *T. reesei* (Zhang et al. 2019) and Basidiomycete fungi (Benocci et al. 2017; Marian et al. 2022), which showed higher expression during growth of *T. reesei* and *P. chrysosporium* on the two plant substrates compared to monosaccharides. The strong species‐specific expression profile of TFs indicates complex and fast‐evolving regulatory networks that governing fungal diverse molecular response to plant biomass.

### 3.3 Metabolomic Profiles Showed Biological Diversity and Industrial Potential

**Figure 6:**
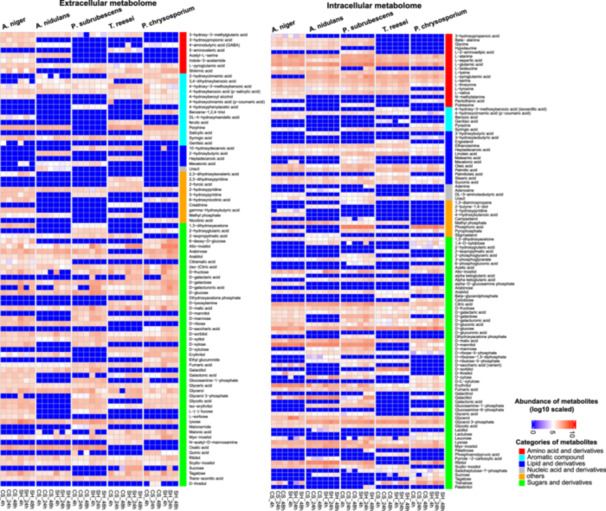
Metabolome profiles of five fungi grown on two crude plant biomass. The abundance of metabolites detected from extracellular media (A) and mycelium (B) are displayed. The average abundance of each tested conditions from low to high is indicated with colors from blue to red. The metabolites are grouped to different categories and highlighted with specific colors. Only the metabolites that show significantly differential abundance in selected comparisons or were uniquely identified in individual species are shown in this figure.

The metabolome profile of fungi grown in the same conditions as transcriptome and proteome analysis was further analyzed. In total, 89 extracellular metabolites and 112 intracellular metabolites (Figure 6 and Supporting Information S2: Table S3) showed differentially profiles across conditions or uniquely presence in one specific species. Most of the selected metabolites belong to sugar, amino acid, nucleic acid, lipid and their derivatives. The sugar‐related compounds account for the largest section of the total detected metabolites for all tested fungi. Many of their abundance profiles not only reflected the composition of the substrate that the fungi grew on, but also partially confirmed the metabolic diversity discussed in the above section and previous studies. The main sugar components of plant biomass, such as d‐glucose, d‐xylose, l‐arabinose, d‐galactose and their metabolic intermediates have been commonly identified in all species (Figure 6). d‐Galacturonic acid showed higher abundance in the extracellular metabolome in SBH than CS for all tested fungi, matching the composition difference between these two substrates. The detection of several sugar derivatives indicates their important role as reserve carbohydrate or adaptation to changing environments (Ocón et al. 2007; Pfyffer et al. 1990), such as trehalose and several polyols (glycerol, erythritol, mannitol, arabitol, ribitol, and inositol) (Figure 6). d‐ribose was not identified in both intracellular and extracellular metabolome for *P. chrysosporium*, but have been detected in four Asomycetes (Figure 6), suggesting different metabolic flux between these two fungal phyla. In addition, xylulose was also uniquely accumulated intracellularly in *P. chrysosporium* (Figure 6B), which could be linked with the lack of l‐xylulose reductase homologs compared to Ascomycete (Figure 4C) (Chroumpi, Peng, Aguilar‐Pontes, et al. 2021). The intracellular accumulation of cellobiose and xylobiose in *P. chrysosporium* (Figure 6B) matches with higher expression of corresponding STs (Figure 3A, such as Phchr2 | 3004542, Phchr2 | 2898453), confirming the important role of these disaccharides in induction of PBD CAZymes for basidiomycetes (Casado López et al. 2018; Hori et al. 2012). One metabolite, 1,3‐diaminopropane, has been uniquely identified in *P. subrubescens*, which is in line with the reported penicillin biosynthesis pathway in *Penicillium chrysogenum* (Esmahan et al. 1994; Martín et al. 2012).

Notably, several high‐value compounds have been identified in the metabolome data during the growth of five fungi on crude plant biomass. For instance, 4‐aminobutyric acid (GABA) was commonly present in samples of *A. niger, A. nidulans* and *P. chrysosporium*. Due to its health benefit, GABA has been widely used as a bioactive compound in the food, pharmaceutical, and animal feed industries (Braga et al. 2024; N. Xu et al. 2017), and the biosynthesis of GABA has attracted increasing interest (Altaib et al. 2022). Compared to extensive studies on bacterial GABA synthesis, fungal GABA production has been poorly studied (N. Xu et al. 2017). The result here suggest GABA could play an essential role for many fungi, such as in oxidative stress tolerance (Coleman et al. 2001). The ortholog of the characterized glutamate decarboxylase in *Aspergillus oryzae* (Kato et al. 2002), that convert l‐glutamate into GABA has also been identified in proteome data all tested fungi (Aspni|11429, Aspnid|10460, Pensub|13426, Trire|112760, Phchr2 | 3001458, Supporting Information S2: Table S2). The detailed biological function of these genes need to be further verified and explored for industrial application. Another value‐added compound 4‐hydroxy‐3‐methoxybenzoic acid (vanillic acid) was broadly identified in the extracellular metabolome of all tested fungi, indicating the industrial potential of bioproduction of this compound by fungi (Lubbers et al. 2021). Lastly, a medical important disaccharide, lactulose (Aït‐Aissa and Aïder 2014) and the vitamin B3 (nicotinic acid), were uniquely identified in the intracellular and extracellular metabolome of *P. chrysosporium, respectively*. Taken together, the identification of these valuable compounds showcases the importance to further explore molecular mechanisms and relevant biotechnology potential of FPBC across diverse fungi species.

## 4 Conclusions

In this study, the transcriptome, proteome, and metabolome of five fungi grown on two crude plant biomass were comparatively analyzed. The gene, protein and metabolites profiles related to plant lignocellulose conversion exhibited strong time‐, substrate‐ and species‐specificity, highlighting the diverse approaches of these fungi for adaptation to different plant biomass and their complex transcription regulation. These findings not only contribute to a deeper understanding of molecular mechanisms of FPBC associated with fungal important ecological roles in the global carbon cycle, but also provide novel insights into better designing fungal cell factories for sustainable production of valuable compounds from renewable plant biomass.

## Clarification

Parts of this work have been submitted in partial fulfillment of the requirements for PhD degree of a co‐author Jiajia Li at Utrecht University. The details of the PhD thesis can be found here, https://dspace.library.uu.nl/handle/1874/430921.

## Author Contributions

**Mao Peng:** conceptualization, writing, methodology, investigation, software, funding acquisition. **Jiajia Li:** investigation. **Li Xu:** investigation; **Tania Chroumpi:** investigation; **Sandra Garrigues:** investigation and writing; **Roland S. Kun:** investigation. **Jiali Meng:** investigation. **Maria Victoria Aguilar‐Pontes:** investigation. **Anna Lipzen:** investigation. **Vivian Ng:** investigation. **Chaevien S. Clendinen:** investigation. **Nikola Tolic:** investigation. **Scott E. Baker:** conceptualization. **Igor V. Grigoriev:** conceptualization, project administration, resources. **Ronald P. de Vries:** conceptualization, writing, supervision, resources, funding acquisition, project administration.

## Ethics Statement

The authors have nothing to report.

## Conflicts of Interest

The authors declare no conflicts of interest.

## Supporting information


**Supplemental Figure 1:** Heatmap showing the expression profile of sugar metabolic genes in each species. **Supplemental Figure 2:** Total numbers of significantly differentially expressed FPBC‐related CAZy, sugar metabolic and transporter genes in transcriptome data (A‐C) and sugar metabolic enzyme in proteome data (D). **Supplemental Figure 3:** Expression profiles of FPBC‐related transcription factors in transcriptome data of five fungi grown on different monosaccharides and crude biomass.

**Supplemental Table 1:** Gene expression values and statistical comparison of transcriptome data of five fungi grown in different conditions. **Supplemental Table 2:** Protein abundance values and statistical comparison of proteome data of five fungi grown in different conditions. **Supplemental Table 3:** Metabolite profiles of five fungi grown in different conditions.

## Data Availability

The data that supports the findings of this study are available in the supporting material of this article. The raw transcriptomics, proteomics, and metabolomics data were deposited in corresponding public databases as described in the materials and methods part.
