# Supplementary material for: Multi‐Omics Analyses Reveal Divergent Molecular Mechanisms Underlying Plant Biomass Conversion by Five Fungi
Source: Microbiologyopen. 2025 Dec 18;14(6):e70201. doi: 10.1002/mbo3.70201 (PMC12714826; doi:10.1002/mbo3.70201)
Supplement: Supplementary file 1 — Supplemental Figure 1: Heatmap showing the expression profile of sugar metabolic genes in each species. Supplemental Figure 2: Total numbers of significantly differentially expressed FPBC‐related CAZy, sugar metabolic and transporter genes in transcriptome data (A‐C) and sugar metabolic enzyme in proteome data (D). Supplemental Figure 3: Expression profiles of FPBC‐related transcription factors in transcriptome data of five fungi grown on different monosaccharides and crude biomass. [file MBO3-14-e70201-s002.pdf]

Supplementary materials

Multi-omics analyses reveal divergent molecular mechanisms underlying plant biomass conversion of five fungi

Mao Peng[1]*, Jiajia Li[1], Li Xu[1], Tania Chroumpi[1], Sandra Garrigues[1], Roland S. Kun[1], Jiali Meng[1], Maria Victoria Aguilar-Pontesa[1], Anna Lipzen[2], Vivian Ng[2], Chaevien S. Clendinen[3], Nikola Tolic[3], Scott E. Baker[4,5], Igor V. Grigoriev[2,6], Ronald P. de Vries[1]

[1] Fungal Physiology, Westerdijk Fungal Biodiversity Institute; Uppsalalaan 8, 3584 CT, Utrecht, The Netherlands

[2] USA Department of Energy Joint Genome Institute, Lawrence Berkeley National Laboratory, 1 Cyclotron Rd, Berkeley, CA 94720, USA

[3]Environmental Molecular Sciences Laboratory, Pacific Northwest National Laboratory, Richland, WA 99354, USA

[4]Microbial Molecular Phenotyping Group, Environmental Molecular Sciences Division, Environmental and Biological Sciences Division, Pacific Northwest National Laboratory, Richland, Washington 99354, USA

[5]DOE Joint BioEnergy Institute, Emeryville, California 94608,USA

[6]Department of Plant and Microbial Biology, University of California Berkeley, Berkeley, CA , USA

*Correspondence: m.peng@wi.knaw.nl

Table of contents:

- Fig. S1: Heatmap showing the expression profile of sugar metabolic genes in each species.
- Fig. S2 Total numbers of significantly differentially expressed FPBC related CAZy, sugar metabolic and transporter genes in transcriptome data and sugar metabolic enzyme in proteome data
- Fig. S3. Expression profiles of FPBC related transcription factors in transcriptome data of five fungi grown on different monosaccharides and crude biomass.

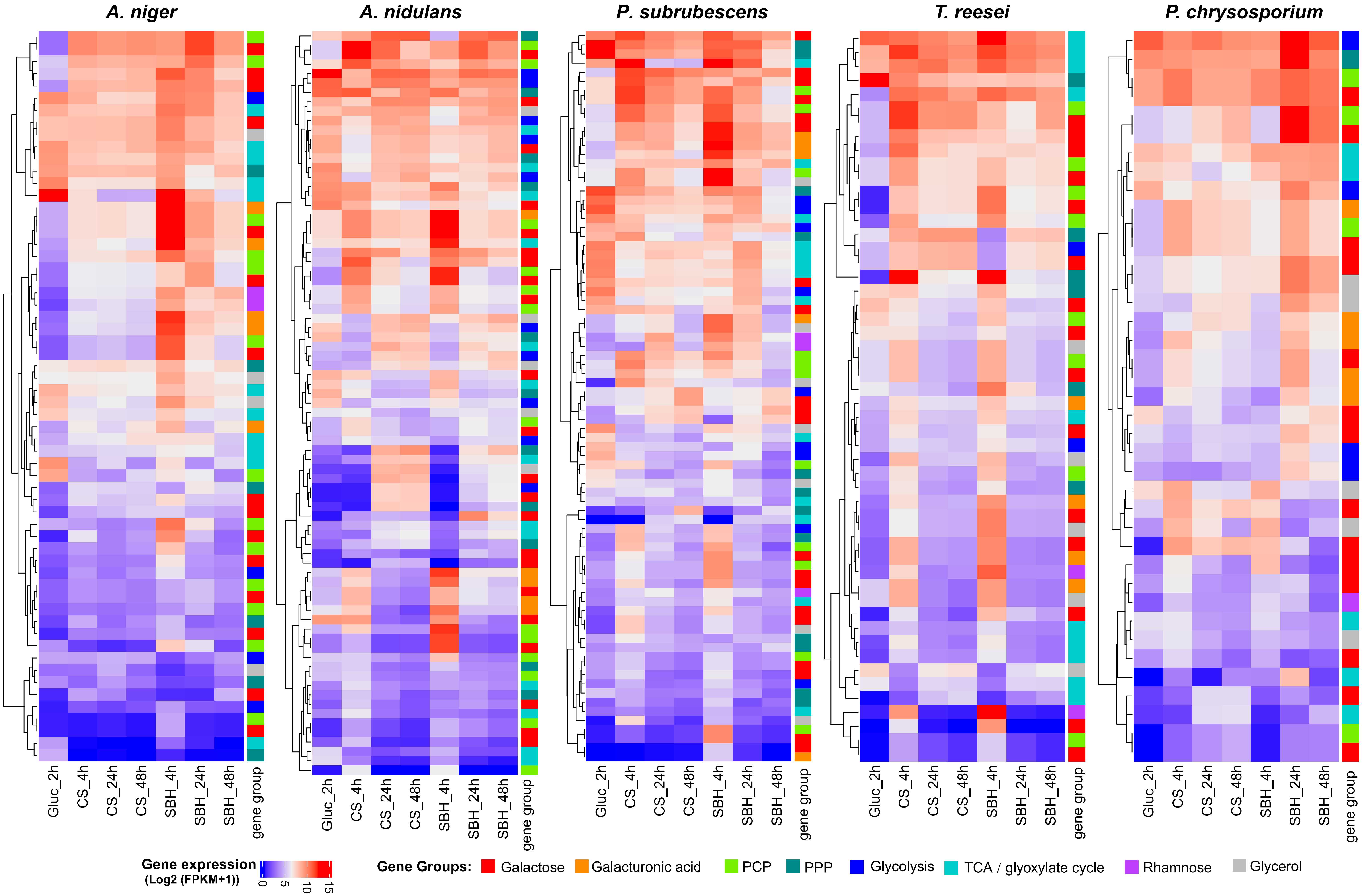

Supplemental Figure 1. Heatmap showing the expression profile of sugar metabolic genes in each species. Expression values from low to high were indicated with color from blue to red.

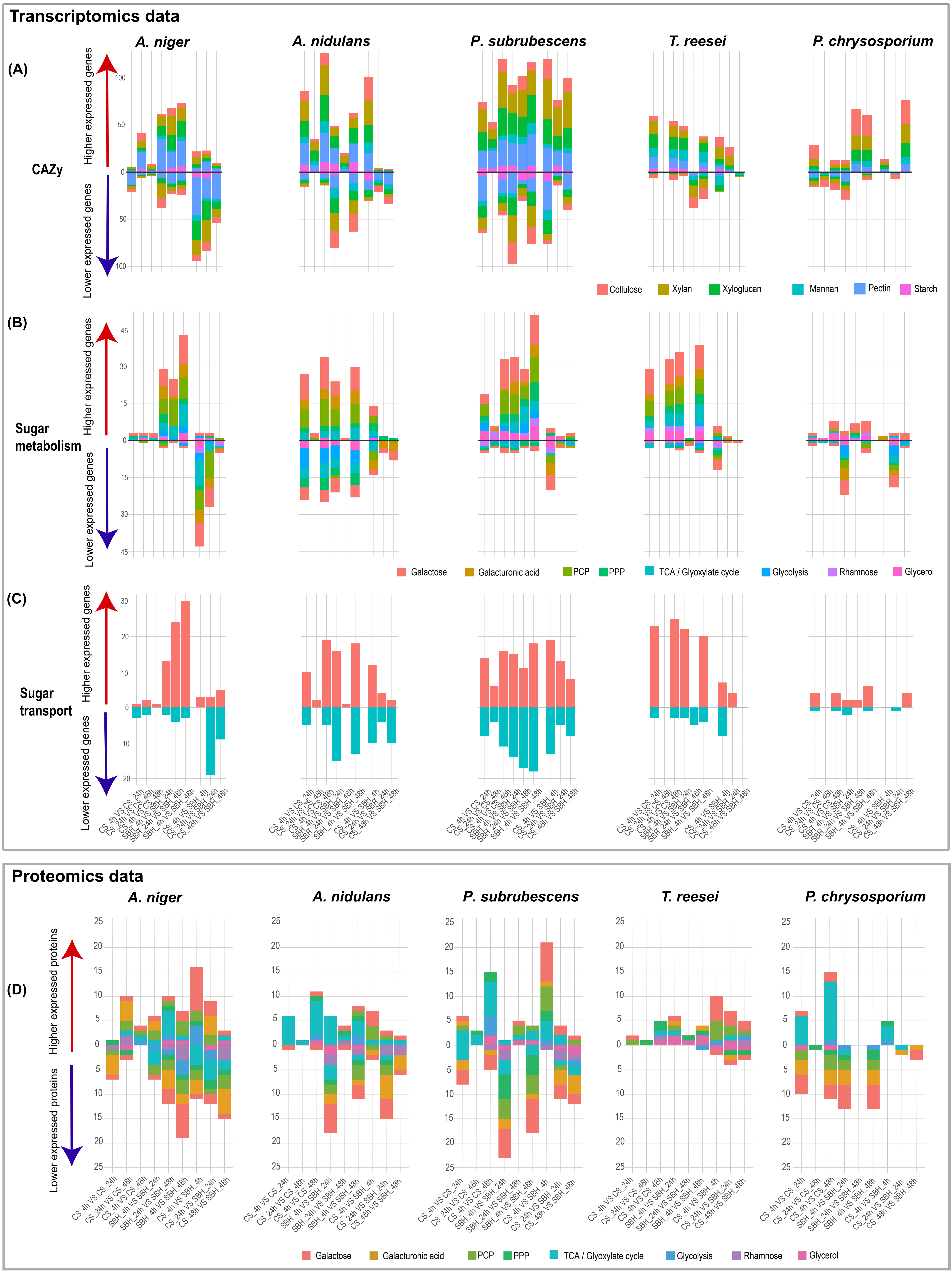

Supplemental Figure 2. Total numbers of significantly differentially expressed FPBC related CAZy, sugar metabolic and transporter genes in transcriptome data (A-C) and sugar metabolic enzymes in proteome data (D).

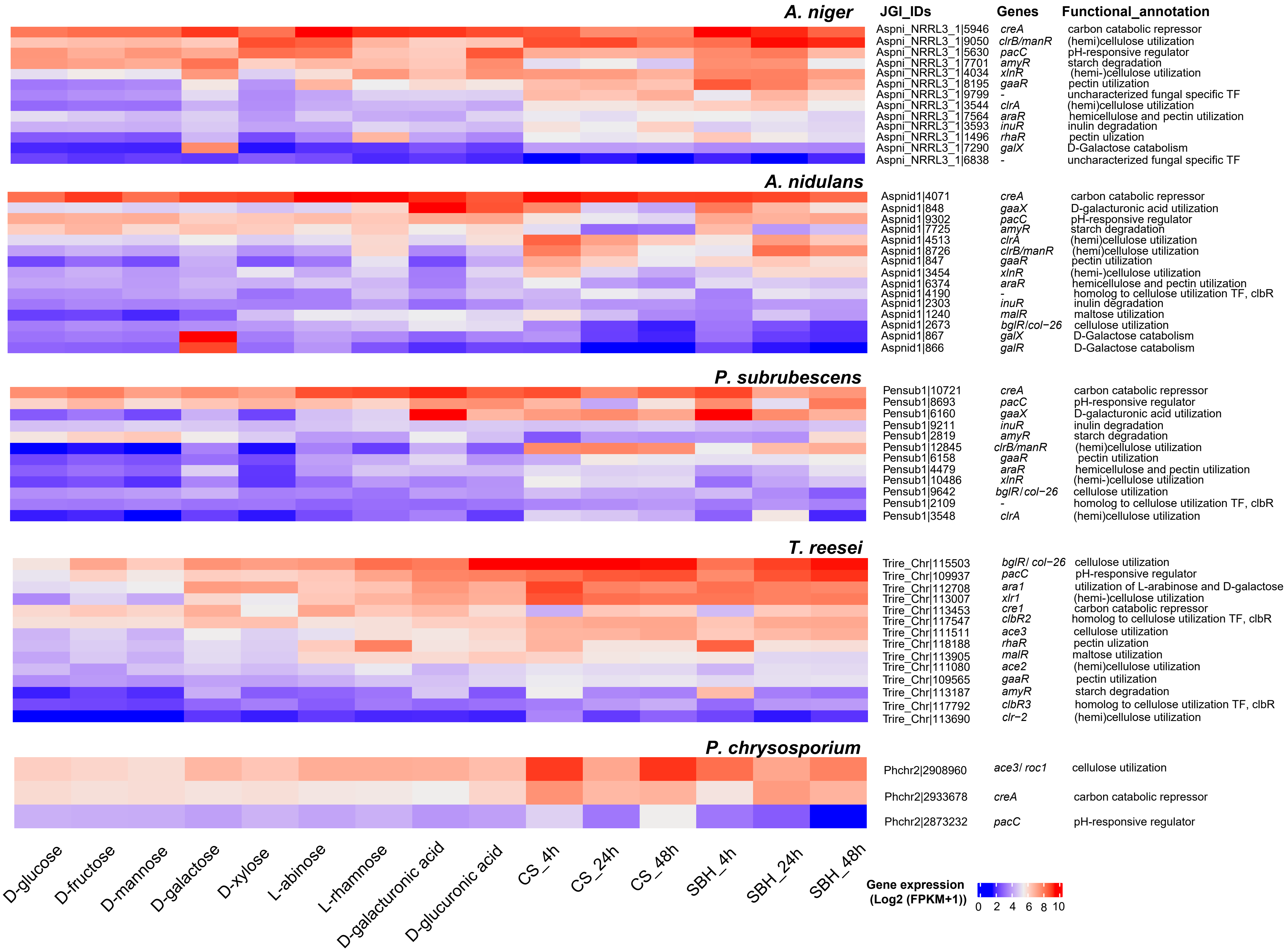

Supplemental Figure 3. Expression profiles of FPBC related transcription factors in transcriptome data of five fungi grown on different monosaccharides and crude biomass.
